# Alpine Adaptive Mechanism on Rhizosphere Microbes Recruitment of *Crepis napifera* (Franch.) Babc. by Multi-Omics Analysis

**DOI:** 10.3390/biology14040345

**Published:** 2025-03-27

**Authors:** Genlin Yang, Weiwei Liu, Xinchun Mo, Zhinan Mei

**Affiliations:** 1 School of Pharmaceutical Sciences, South-Central Minzu University, Wuhan 430074, China; ygl517258@163.com; 2 Forestry Institution of Science of Yunnan Lijiang, Lijiang 674199, China; 3 Lijiang Forest Biodiversity National Observation and Research Station, Kunming Institute of Botany, Chinese Academy of Sciences, Kunming 650201, China; liuweiwei@mail.kib.ac.cn; 4 School of Applied Technology, Lijiang Normal University, Lijiang 674199, China; 5 College of Plant Science and Technology, Huazhong Agricultural University, Wuhan 430070, China

**Keywords:** *Crepis napifera* (Franch.) Babc., multi-omics analysis, rhizosphere microbes recruitment, terpene biosynthesis

## Abstract

**Background:** The accumulation of secondary metabolites in medicinal plants is often influenced by a variety of factors, and rhizosphere microorganisms typically engage in complex interactions with their host plants. *Crepis napifera* (Franch.) Babc., a regionally significant medicinal plant, contains a diverse array of terpenoids and demonstrates substantial potential for resource development and utilization. **Methods:** Transcriptome sequencing, metabolomic profiling, and 16S rRNA gene amplicon sequencing were employed to assess the transcriptional expression patterns, metabolic variations, and rhizosphere microbial community composition of *C. napifera* (Franch.) Babc. roots distributed across various regions. **Results:** A total of 3679, 8615, and 11,333 differentially expressed genes (DEGs) were identified in the pairwise comparisons between H1 vs. H2, H2 vs. H3, and H1 vs. H3, respectively. Notably, 497 DEGs were consistently detected across all three comparisons. Additionally, Weighted Gene Co-expression Network Analysis (WGCNA) revealed that the expression levels of genes within the turquoise and yellow modules exhibited a significant positive correlation with elevation. In total, 462 differentially expressed metabolites (DEMs) were identified across the same comparisons. Among these compounds, terpenoids, phenolic acids, amino acids and their derivatives, lipids, and alkaloids accounted for 62.98% of the total differential metabolite content. The accumulation patterns of DEMs varied significantly across different regions in the roots of *C. napifera* (Franch.) Babc. under the three altitude conditions. In response to environmental conditions and the survival strategy of *C. napifera* (Franch.) Babc. in alpine areas, an investigation into the rhizosphere microbial community was conducted. Four key microbial genera were identified as being correlated with terpenoid biosynthesis and plant nutritional metabolism. Specifically, *Pedosphaera*, *Acidothermus*, and *Nevskia* exhibited terpene biosynthesis capabilities. Additionally, *Herbaspirillum*, a common microorganism involved in plant nitrogen fixation, respiration, carbon metabolism, and cell wall metabolism, was also enriched in the rhizosphere of *C. napifera* (Franch.) Babc. These findings suggested that *C. napifera* (Franch.) Babc. might recruit these microorganisms to enhance its resistance to environmental stress in alpine areas. **Conclusions:** The accumulation of terpene in *C. napifera* (Franch.) Babc. across different regions was influenced by transcriptional changes. The rhizosphere microbial communities also changed during this process, showing a recruitment effect that enhances plant growth and offers potential value.

## Simple Summary

*Crepis napifera* (Franch.) Babc., a terpene-rich plant belonging to the Asteraceae family, is indigenous to Southwestern China. This species has received limited research attention and remains largely unknown to the public. In this study, we performed a comprehensive evaluation of the medicinal root of *C. napifera* (Franch.) Babc. by examining its transcriptional expression profiles, metabolite accumulation characteristics, and rhizosphere microbial communities across various regions. Through combined multi-omics analysis, distinct microbial recruitment patterns were observed in the rhizosphere of *C. napifera* (Franch.) Babc. Notably, four key symbiotic bacterial genera were identified in the roots of *C. napifera* (Franch.) Babc., namely *Pedosphaera*, *Acidothermus*, *Nevskia*, and *Herbaspirillum*. The first three genera were found to exhibit terpene biosynthesis capabilities at higher altitudes. Genus *Herbaspirillum*, a widely distributed bacteria, was found to involve in a variety of physiological functions within *C. napifera* (Franch.) Babc. in alpine areas by functional diversification analysis. Our research has provided valuable insights into the accumulation characteristics of bioactive compounds and the recruitment mechanisms of specific microbial communities, thereby laying a solid foundation for the future development and utilization of this plant resource.

## 1. Introduction

*Crepis napifera* (Franch.) Babc., a prominent member of the Cichorioideae subfamily within the Asteraceae family, is esteemed in traditional folk medicine for its diverse and abundant chemical constituents in the northwest of Yunnan Province, China. It is utilized by the Naxi, Bai, Yi, and other ethnic groups for the treatment of digestive and respiratory ailments. The therapeutic potential of the genus *Crepis* has been validated by extensive research, highlighting its efficacy in promoting health and addressing various medical conditions [1]. Owing to its antioxidant, anti-inflammatory and hypolipidemic activity, *C. napifera* (Franch.) Babc. is regarded as having potential in the prevention of cardiac diseases [2]. Notable compounds include friedelin, *β*-sitosterol, stigmasterol, chlorogenic acid, and a variety of flavonoids, all of which are renowned for their significant medicinal properties and minimal adverse effects [3,4,5]. Nevertheless, numerous challenges remain in elucidating the biosynthetic mechanisms of active compounds, transcriptional regulation patterns of associated terpene biosynthesis, metabolite accumulation characteristics, and rhizosphere microflora-related survival strategies of this medicinal plant within alpine environments. Hence, in addition to its medicinal value, comprehending the ecological adaptability of *C. napifera* (Franch.) Babc. to a variety of environmental conditions, particularly those associated with altitude, holds considerable significance.

High-altitude environments are characterized by intense solar and UV radiation, prolonged exposure to sunlight, low temperatures, and substantial diurnal temperature variations, as well as low humidity. These environmental stressors can significantly impact physiological processes and result in notable alterations in plant morphology and metabolism, especially in alpine areas [6,7,8,9,10]. A significant accumulation of secondary metabolites occurred in medicinal plants under harsh natural environments at high altitudes, and the accumulated patterns of the metabolites were associated with the altitude change [11,12]. They synthesized a diverse array of secondary metabolites, comprising various phytochemicals that were essential for their growth, defense mechanisms, and adaptability. These bioactive compounds are frequently employed in traditional healthcare practices and can be modulated by environmental factors such as altitude [13,14,15]. With increasing altitude, salidroside phenol was accumulated extensively in the roots and rhizomes of *Picrorhiza kurroa* [16]. Additionally, differentially expressed genes (DEGs) associated with high-altitude adaptation in *Potentilla bifurca* were predominantly involved in the biosynthesis of secondary metabolites, including sesquiterpenoids, triterpenoids, and flavonoids [17]. In *Herpetospermum pedunculosum*, the secondary metabolite Tricetin was found to be significantly upregulated with the increasing altitude [18]. In *Rhodiola crenulata*, compounds such as l-hyoscyamine, 5,7-dihydroxychromone, and quillaic acid exhibited differential expression patterns with the altitude change [19]. However, there is a notable lack of research specifically addressing how the morphology and secondary metabolite profiles of *C. napifera* (Franch.) Babc. vary across different altitudes.

This study systematically evaluated *C. napifera* (Franch.) Babc. populations across various regions through an integrative analysis of transcriptional regulation, metabolite accumulation, and rhizosphere microbial diversity. Specifically, we identified the genes and metabolites that were differentially expressed or accumulated under these conditions, with particular emphasis on terpenoid accumulation among secondary metabolites. We hypothesized that environmental changes would lead to higher terpenoid accumulation in *C. napifera* (Franch.) Babc. populations at higher altitudes, as well as induce shifts in rhizosphere microbial communities. The aim of this study is to employ multi-omics approaches to comprehensively assess the growth adaptability of *C. napifera* (Franch.) Babc. in different regions, thereby providing a scientific foundation for the exploration and sustainable utilization of this valuable medicinal plant resource.

## 2. Materials and Methods

### 2.1. Plant Materials, Morphological Investigation, and Soil Characteristics

*C. napifera* (Franch.) Babc. samples were collected at three different altitudes: the low-altitude group (1858 m, H1, Jin Kedu, Xingming Xingyuan Village, Longpan Township, Yulong County, Yunnan Province, China. E: 100°04′25.7829″, N: 26°59′53.3842″), the medium-altitude group (2417 m, H2, Le Shibanman, Xingwen Qiping Village, Longpan Township, Yulong County, Yunnan Province, China. E: 100°05′18.0637″, N: 26°45′50.7415″), and the high-altitude group (2893 m, H3, Jizi Village, Tai’an Township, Yulong County, Yunnan Province, China, E: 100°05′18.0637″, N: 26°45′50.7415″). For each *C. napifera* (Franch.) Babc. habitat, the environmental factors are listed at Table 1. Root samples of 3-year-old plants from three altitudes were collected in December, 2024, ensuring at least three biological replicates for each group. As soon as the samples were collected, they were flash-frozen in liquid nitrogen and kept at −80 °C for subsequent analysis.

A total of 15 fresh individuals collected from three altitudes were morphologically investigated in each sample site. The stem lengths, average fresh weight, pH value, total carbon content (g/cm^3^), and density of roots from three locations were measured. Soils for this study were collected during winter 2024 from three altitudes mentioned at previous sample collection sites. Three soil cores (0–10 cm depth) were randomly sampled at each sample site. To obtain a representative rhizosphere microbial community, soil samples from each site were combined, homogenized, and maintained at cold temperatures in the field until they were prepared in the laboratory. One fraction was immediately frozen at −80 °C for microbial analysis. The other fraction was kept at 4 °C for 1 day before conducting the soil physical and chemical properties assay, and the soil characteristics from different sample sites are listed at Table 2.

### 2.2. Transcriptome Sequencing and Differential Gene Expression Analysis

For the root tissues of *C. napifera* (Franch.) Babc. collected from three different altitude levels, total RNA extraction was carried out. First, the frozen roots were grounded in liquid nitrogen. Subsequently, Trizol reagent was employed for RNA extraction. The purity and concentration of the extracted RNA were evaluated with a NanoDrop 2000 spectrophotometer (Thermo Fisher Scientific Co. Ltd., Waltham, MA, USA). Additionally, quality control was further confirmed through agarose gel electrophoresis and RNA integrity assessment using an Agilent 2100 Bioanalyzer (Agilent Technologies Inc., Beijing, China). A total of 5 µg of RNA per sample, meeting the criteria of a concentration of at least 200 ng/µL and an OD260/280 ratio ranging from 1.8 to 2.2, was utilized as the input material for the library preparation. RNA purification was performed using Oligo (dT), followed by cDNA synthesis and amplification with the Truseq™ RNA sample preparation kit (Illumina Inc, San Diego, CA, USA). Finally, the cDNA library was quantified using the TBS380 system and sequenced on the Illumina NovaSeq 6000 platform (Illumina Inc, San Diego, CA, USA).

Raw reads were initially processed with Trimmomatic (version 0.38) to eliminate low-quality sequences. Sequences with ambiguous nucleotides exceeding 10%, quality values lower than 20, and lengths shorter than 50 bp were discarded [20]. After this quality control step, the clean reads were utilized for de novo transcriptome assembly employing Trinity (version 2.14.0) [21]. Subsequently, gene-level read counts were employed to identify differentially expressed genes (DEGs) by DESeq2 package (version 1.30.1) [22].

### 2.3. Metabolite Extraction and Analysis via UPLC-MS/MS

A lyophilizer (Scientz-100F) (Ningbo Xinzi Biotech Co., Ltd., Ningbo, Zhejiang, China) was used to pretreat the samples. Subsequently, a MM400 grinder (Verder Scientific Headquarters Inc., Haan, Germany) operating at a frequency of 30 Hz for 1.5 min was used to grind the samples into powder. The sample powder was then mixed with methanolic aqueous internal standard extract and thoroughly vortexed. After centrifugation, the supernatant was collected and filtered to prepare for the UPLC-MS/MS analysis.

The analysis was performed using a UPLC-ESI-MS/MS system. Tandem mass spectrometry (MS/MS) was employed under multiple reaction monitoring (MRM) conditions, with nitrogen set as the collision gas at medium pressure. Specific MRM transitions were monitored in accordance with the elution profiles of the metabolites.

For the analysis of differential metabolites between groups, the criteria of variable importance in projection (VIP > 1) and absolute log2 fold change (|Log2FC| ≥ 1.0) were utilized. VIP values were extracted from the results of the orthogonal partial least squares discriminant analysis (OPLS-DA). The MetaboAnalystR package in R was used to generate core plots and permutation plots [23]. Prior to the data of OPLS-DA, the result was log-transformed (log2) and mean-centered.

### 2.4. Rhizosphere and Soil Microbial DNA Extraction and 16S rRNA Sequencing

Initially, samples were rinsed with ddH_2_O. Subsequently, centrifugation was carried out at 2350× *g* for 10 min to collect the sediment from the sample. This sediment was treated as rhizosphere microbial samples. For both environmental soil (S) and rhizosphere soil (RS) samples, microbial DNA was extracted using the HiPure Soil DNA Kit B (Guangzhou Magen Biotechnology Co., Ltd., Guangzhou, China). The concentration of DNA was quantified using the Qubit dsDNA HS Assay Kit (Verder Scientific Headquarters, Haan, Germany). Before sequencing on the Illumina MiSeq platform, the library concentration was validated with a Qubit 3.0 fluorometer (Verder Scientific Headquarters, Haan, Germany).

The raw data were subjected to quality filtering using Mothur (version 1.39.5) [24]. Specifically, reads that contained ambiguous base calls (denoted as “N”), along with those shorter than 300 bp or longer than 500 bp, were eliminated. The resulting clean reads were then employed for taxonomic analysis via the QIIME pipeline [25]. Operational taxonomic units (OTUs) were clustered at a 97% sequence similarity threshold. The QIIME’s summarize_taxa function was utilized to determine the microbial composition at each taxonomic level [25]. The alpha diversity of the microbial communities in the R and RS samples was evaluated using the alpha_rarefaction.py script, and diversity matrices were computed with the vegan R library. Additionally, linear discriminant analysis effect size (LEfSe) was utilized to analyze differentially abundant taxonomical features among the microbial communities. This analysis aimed to identify significant taxonomical biomarkers with a *p*-value from the Kruskal–Wallis test.

### 2.5. Species Abundance and Diversity Analysis

The Micreco package was employed to conduct an analysis of alpha and beta diversities, as well as species abundances, among different samples [26]. The alpha diversity metrics, namely Chao1, ACE, Shannon Diversity Index, and Simpson Diversity Index, serve as indicators that reflect the species richness and diversity within a community. At the genus level, the “deseq2” package version 1.42.1 within the R programming environment is utilized to analyze and determine the differences that exist between the samples of each group [22]. Bacteria with log2 fold change values exceeding 0.7 or less than −0.7 and adjusted *p*-values less than 0.05 were regarded as significantly different.

### 2.6. Principal Component and Pearson Correlation Analysis

Unit variance scaling was applied to preprocess the data for each metabolite or microorganism across all samples. The prcomp function available in R was utilized to carry out the principal component analysis (PCA). The cor function in R was employed to calculate correlation coefficients. Significance testing of the correlations was conducted using the corPvalueStudent function from the WGCNA package in R [27]. Mfuzz trend analysis was performed on differential metabolites and differential bacteria [28]. Selected metabolites and bacteria exhibiting an increasing trend from H1 to H2 to H3 were subjected to the Pearson correlation analysis, filtering for *p* < 0.05 and a correlation coefficient greater than 0.5.

### 2.7. KEGG Annotation and Enrichment Analysis

The identified metabolites were annotated by referring to the KEGG Compound database. After annotation, these metabolites were mapped to the KEGG Pathway database [29]. Metabolite sets enrichment analysis (MSEA) was conducted to analyze the pathways that contained significantly regulated metabolites [30].

### 2.8. RNA Isolation and Quantitative Real-Time PCR

RNA isolation and cDNA reverse transcription were performed using MonScript™ RTIII All-in-One Mix with dsDNase (MR05101, Monad, Suzhou, China). The QuantiNova SYBR Green PCR Kit (208054, QIAGEN, Beijing, China) was utilized for qRT-PCR. Actin1 was set as the internal reference. qRT-PCR data were analyzed through the 2^−ΔΔT^ method, and statistical differences were determined via one-way ANOVA using results from three biological replicates [31]. All primers are provided in Table S1.

## 3. Results

### 3.1. Phenotypic Analysis of C. napifera (Franch.) Babc. Under Varying Altitude Conditions

**Figure 1:**
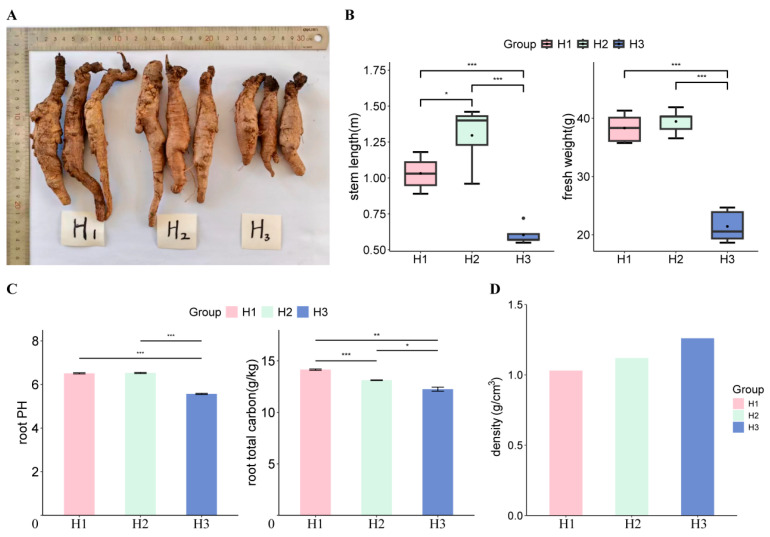
Morphological identification of *C. napifera* (Franch.) Babc. in different altitude conditions. (**A**) Phenotypic changes in *C. napifera* (Franch.) Babc. under different altitude conditions. (**B**) Stem length (cm) and fresh weight (g) under different altitude conditions. * indicated significance between two groups; *** indicated extremely significant between two groups. (**C**) pH value and total carbon content (g/cm^3^) of roots under different altitude conditions. * indicated significance between two groups; ** indicated very significant between two groups; *** indicated extremely significant between two groups. (**D**) Density of the roots at different altitudes. H1, roots of *C. napifera* (Franch.) Babc. collected from a low altitude (1858 m); H2, roots of *C. napifera* (Franch.) Babc. collected from a medium altitude (2417 m); H3, roots of *C. napifera* (Franch.) Babc. collected from a high altitude (2893 m).

The phenotypic characteristics of *C. napifera* (Franch.) Babc. exhibited notable changes when subjected to different altitude conditions (Figure 1A and Table S2). Specifically, the stem length in the H2 group was significantly greater than that in the H1 group, while the H3 group showed a marked reduction in stem length compared to both H1 and H2 (Figure 1B and Table S2). In terms of fresh weight, the H3 group also had significantly lower weights than both H1 and H2 (Figure 1B and Table S2).

We measured the pH levels of the roots across the three altitude groups and found no significant differences between H1 and H2. However, the H3 group displayed a significantly lower pH compared to both H1 and H2 (Figure 1C and Table S2). Additionally, the root density was increased with the altitude rising, suggesting an adaptive response to environmental stressors at higher elevations (Figure 1D and Table S2). Collectively, these results indicated that, under varying altitude conditions, the phenotypic traits of *C. napifera* (Franch.) Babc. exhibited significant differences.

### 3.2. Transcriptome Analysis of C. napifera (Franch.) Babc. Under Varying Altitude Conditions

**Figure 2:**
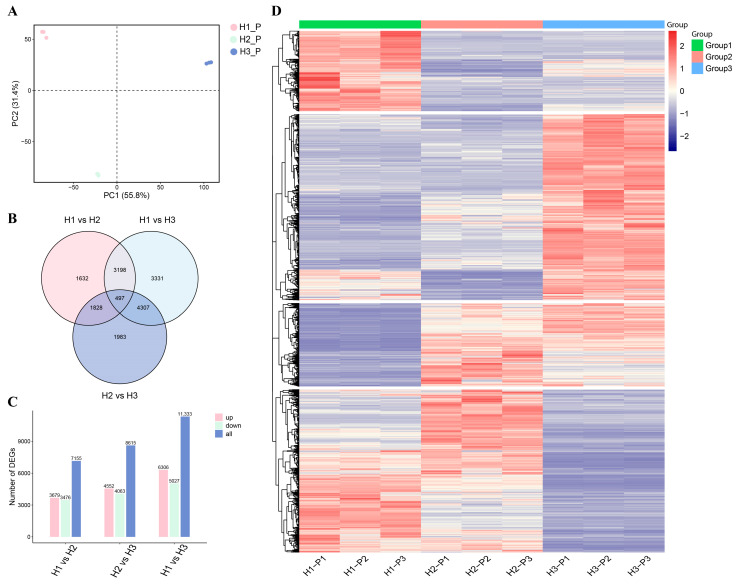
Transcriptome analysis of the roots of *C. napifera* (Franch.) Babc. at different altitudes. (**A**) Principal component analysis (PCA) of the RNA-Seq data. (**B**) Venn diagram of the DEGs. (**C**) The number of genes that were up- and downregulated under different altitude conditions. (**D**) Heatmaps of the DEGs compared between different groups.

To further investigate the molecular responses of *C. napifera* (Franch.) Babc. to altitude, RNA sequencing was performed on root tissues, and the information on quality control is shown in Table S3. The principal component analysis (PCA) revealed clear clustering of the three groups, indicating that their transcriptional profiles were distinctly influenced by altitude (Figure 2A). A total of 3679, 8615, and 11,333 differentially expressed genes (DEGs) were identified in the H1 vs. H2, H2 vs. H3, and H1 vs. H3 comparisons, respectively (Figure 2B,C). Notably, 1632 DEGs were unique to the H1 vs. H2 comparison, while 3331 were unique to H2 vs. H3, and 1986 were unique to H1 vs. H3. Furthermore, 497 DEGs were common across all comparisons (Figure 2B).

The analysis showed that 3679, 4552, and 6306 DEGs were upregulated in the H1 vs. H2, H2 vs. H3, and H1 vs. H3 comparisons, respectively, while 3479, 4063, and 5027 DEGs were downregulated (Figure 2C and Table S4). The WGCNA showed that the expression levels of the genes in the turquoise and yellow modules increased with the elevation (Figure S1). In these modules, many genes associated with terpene synthesis, including MAS and CYP76AH1, exhibited significantly higher expression levels in the H3 group (Figure 2D and Table S5).

### 3.3. Metabolome Analysis of C. napifera (Franch.) Babc. Under Varying Altitude Conditions

**Figure 3:**
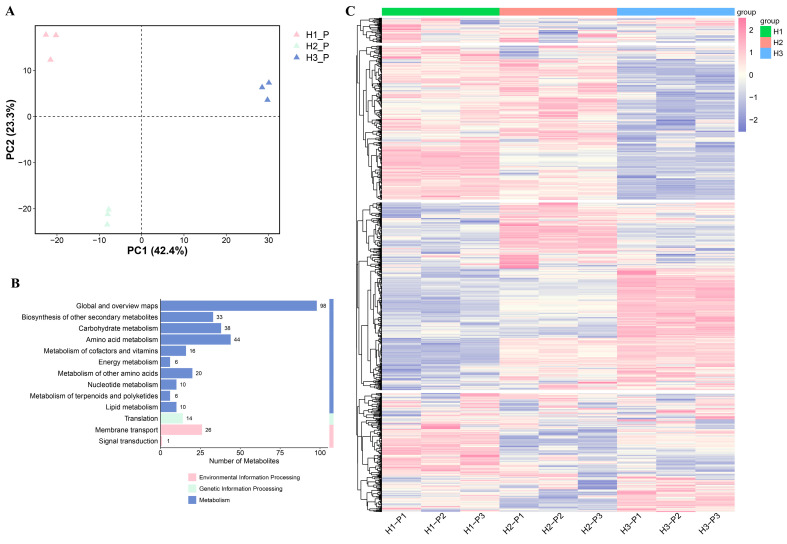
Metabolomics analysis of the roots of *C. napifera* (Franch.) Babc. at different altitudes. (**A**) Principal component analysis (PCA) of the metabolome. (**B**) KEGG analysis of the metabolomics. (**C**) Heatmaps of the DEMs compared between different groups.

Metabolomic analyses were conducted to characterize the metabolic profiles of *C. napifera* (Franch.) Babc. roots. PCA revealed that a distinct clustering of replicates was observed within each group, with the model accounting for 65.7% of the total variance (Figure 3A). In total, 462 differentially expressed metabolites (DEMs) were identified in the H1 vs. H2, H2 vs. H3, and H1 vs. H3 comparisons (Figure S2A). Among these compounds, terpenoids, phenolic acids, and amino acids and their derivatives, as well as lipids and alkaloids, exhibited the highest contents, accounting for 62.98% of the total content of the differential metabolites (Figure S2B). Based on the trends observed in metabolomic accumulation, three distinct clusters were identified. There are notable variations in the accumulation of metabolites in the roots of *C. napifera* (Franch.) Babc. across different regions (Figure S2C).

KEGG pathway enrichment analysis revealed that these DEMs were predominantly engaged in terpenoid and polyketide metabolism, the biosynthesis of secondary metabolites, and carbohydrate metabolism. Moreover, the pathways associated with genetic information processing, for instance, translation, as well as environmental information processing, such as membrane transport and signal transduction were also found to be enriched (Figure 3B and Table S6). The heatmap visualization revealed differential accumulation patterns of metabolites in the roots of *C. napifera* (Franch.) Babc. from the three altitude conditions. It can be observed that the cumulative patterns of DEMs vary across different regions (Figure 3C).

### 3.4. Bacterial Diversity Analysis of Roots of C. napifera (Franch.) Babc. Under Varying Altitude Conditions

**Figure 4:**
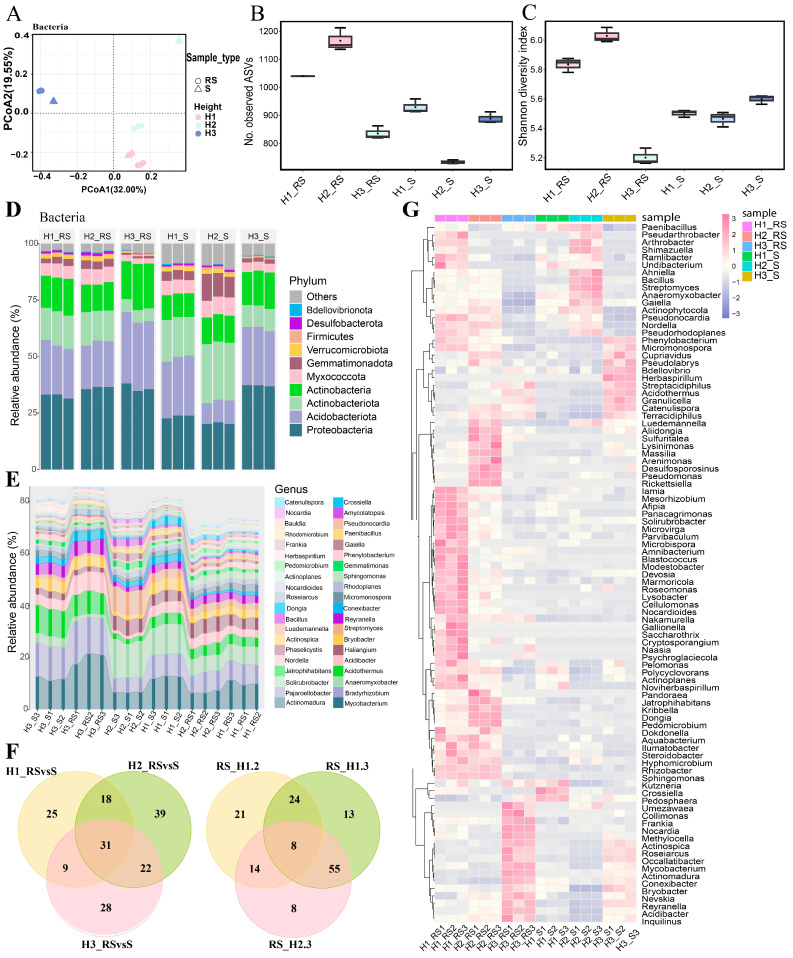
Analysis of the bacteria community from roots of *C. napifera* (Franch.) Babc. at different altitudes. (**A**) Principal component analysis (PCA) of the detected root (R) and rhizosphere soil (RS) samples via 16s rRNA sequencing. (**B**) Display of the observed amplicon sequence variants (ASVs) in R and RS. (**C**) The Shannon Diversity Index of R and RS. (**D**) Relative abundance of R and RS at the phylum level. (**E**) Relative abundance of R and RS at the genus level. (**F**) Venn diagram depicting the relative abundance of R and RS at the genus level. (**G**) Heatmap of the abundance of R and RS at the genus level.

To examine the influence of altitude on the rhizosphere microbial communities associated with *C. napifera* (Franch.) Babc., we performed 16S rRNA sequencing on rhizosphere and root samples, and the information on quality control is shown in Table S3. The PCA demonstrated distinct clustering among the groups, indicating that altitude significantly influences microbial community composition (Figure 4A). The H2_RS group exhibited the highest number of observed amplicon sequence variants (ASVs), while the H2_S group had the lowest (Figure 4B). The Shannon Diversity Index showed that H2_RS had the greatest microbial diversity, whereas H3_RS exhibited the lowest (Figure 4C).

At the phylum level, dominant groups such as *Actinobacteria*, *Proteobacteria*, and *Acidobacteriota* were detected across all samples (Figure 4D). At the genus level, taxa including *Actinoplanes*, *Actinomadura*, *Amycolatopsis*, and *Streptomyces* were present in all groups (Figure 4E and Figure S3). Unique microorganisms were identified in each altitude comparison, with 25, 39, and 28 unique taxa found in the H1_RS vs. S, H2_RS vs. S, and H3_RS vs. S comparisons, respectively, while 31 taxa were common across all comparisons (Figure 4F). Notably, the microbial communities exhibited distinct enrichment patterns at different altitudes, with genera such as *Actinospica*, *Roseiarcus*, *Occallatibacter*, and *Mycobacterium* showing a higher abundance in both H3_RS and H3_S groups (Figure 4G).

### 3.5. Multi-Omics Data Reveal Genes and Microbial Communities Related to the Metabolism of Terpene Compounds

**Figure 5:**
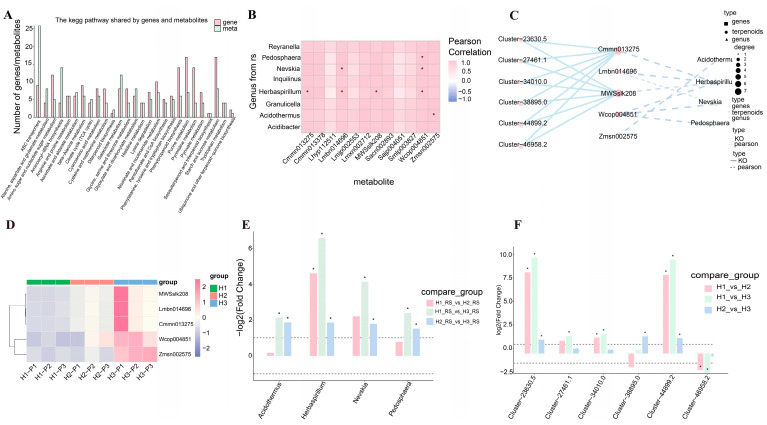
The potential correction on gene expression, metabolites, and microorganisms at different altitudes. (**A**) The KEGG both enriched in RNA and metabolites detection. (**B**) The Pearson’s correlation between detected genera and metabolites. The symbol “*” represented the metabolites showed correlation with microbe. (**C**) Correlation analysis of the transcriptome, metabolome, and 16S sequencing. (**D**) Analysis of the accumulation pattern of the above metabolites. (**E**) Comparative analysis of the gene expression levels under different altitudes comparison. The symbol “*” represented the significant differentiation with *p* value < 0.05. (**F**) Comparative analysis of the microbial community’s enrichment under comparison of different altitudes. The symbol “*” represented the significant differentiation with *p* value < 0.05.

To further investigate the interrelations among gene expression, metabolite profiles, and microbial communities, we conducted correlation analyses. Pathways associated with diterpenoid, sesquiterpenoid, and triterpenoid biosynthesis, as well as starch and sucrose metabolism, were enriched in both RNA-seq and metabolomic analyses (Figure 5A and Figure S4). The expression of genes involved in the synthesis of terpenoid metabolites such as Momilactone A and Ferruginol was significantly elevated in the H3 samples. Concurrently, higher concentrations of kaurenic acid and isopimaric acid were detected in the H3 group, alongside increased expression of the upstream gene GA3, which is critical for kaurenic acid synthesis (Figure 5B).

Pearson correlation analysis revealed strong associations between specific microbial genera and metabolites. For example, *Pedosphaera* was strongly correlated with Wcop004851, while *Nevskia* was correlated significantly with both Lmbn014696 and Wcop004851. Additionally, *Herbaspirillum* showed substantial correlations with various metabolites, indicating its potential role in the ecological dynamics of the rhizosphere (Figure 5C).

As the altitude increased, the accumulation patterns of these metabolites shifted significantly, with enhanced levels of MWSslk208, Zmsn002575, Cmmn013275, Lmbn014696, and Wcop004851 observed in the H3 group compared to H1 and H2 (Figure 5D). The microbial communities also displayed varied enrichment patterns at different altitudes. Notably, *Herbaspirillum* showed significantly higher enrichment in the H1_RS vs. H2_RS, H1_RS vs. H3_RS, and H2_RS vs. H3_RS comparisons, while genera such as *Acidothermus*, *Nevskia*, and *Pedosphaera* exhibited greater enrichment in the H1_RS vs. H3_RS and H2_RS vs. H3_RS comparisons (Figure 5E). In these processes, many genes may play important roles. The expression level of many genes, such as Cluster-27461.1, Cluster-34010.0, and Cluster-44899.2, increase with the altitude, while the expression level of Cluster-46958.2 was downregulated in the same condition (Figure 5F).

**Figure 6:**
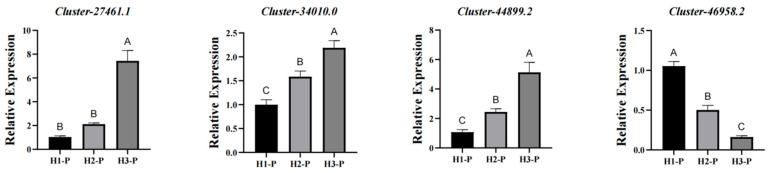
The expression patterns of selected genes under altitudinal conditions were evaluated using qRT-PCR. Error bars represent the standard deviations (SD) of the means (with *n* = 3). The letters A, B, and C indicate substantial differences in expression among the groups. If the letters are identical, it signifies no significant difference between the groups.

To validate the reliability of the transcriptome data, four genes mentioned in Figure 5F were selected to analyze their expression levels via qRT-PCR. The qRT-PCR analysis revealed a significant correlation between the relative expression of these genes as the altitude rises and the transcriptome information, thereby indicating the trustworthiness of the data (Figure 6).

## 4. Discussion

High-altitude environments are characterized by distinct conditions, including significant diurnal temperature variations, reduced oxygen levels, low temperatures, and heightened ultraviolet radiation. For instance, research has uncovered that the total anthocyanin and vitamin C content in the fruits of *Fragaria* × *ananassa* cultivated at higher altitudes was lower than in those grown at lower elevations [31]. As a result, plants native to these regions exhibited various adaptive strategies to survive and thrive [32]. Additionally, notable alterations in fruit coloration have been observed in peaches from the Qinghai–Tibet Plateau, with substantial differences in carotenoid accumulation between Tibetan and other cultivated areas [33]. In the peel of *Malus domestica* grown at high altitudes, the anthocyanin and phenolic compound contents were observed to increase, associated with altitude rising [9]. Similarly, *Prunus persica* was found to exhibit varying antioxidant capacities when grown under different altitude conditions [10]. The influence of altitude on flavonoid biosynthesis in pigmented potatoes (*Solanum tuberosum* L.) was also documented, where red and purple tubers from high altitudes contained higher flavonoid levels than those with darker flesh from lower altitudes [34]. Furthermore, the growing conditions at different altitudes had been shown to affect the quality of medicinal plants. For example, in *Cyclocarya paliurus*, flavonoid accumulation in leaves varied significantly with altitude [35]. In the case of *Codonopsis pilosula*, the triterpene levels were more pronounced at higher elevations [10]. Despite these findings, there was limited research on how varying altitudes influence metabolites in *C. napifera* (Franch.) Babc.

In this research, significant differences in transcriptional levels and variations in metabolite accumulation were observed in *C. napifera* (Franch.) Babc. populations distributed across different altitudinal regions. In the interaction with soil microorganisms, *Pedosphaera* not only enhanced the fertility of the plant rhizosphere soil by improving phosphorus and potassium absorption but was also intimately involved in the biosynthesis of terpenoids such as (-)-loliolide, which was consistent with our findings. As *C. napifera* (Franch.) Babc. distributed to higher altitudes, the enrichment of *Pedosphaera* and the synthesis of terpenoids were interrelated [36,37]. Furthermore, it has been proven that *Acidothermus* is involved in the biosynthesis of triterpenoids and assists plants in coping with environmental stress, which aligns with our findings in *C. napifera* (Franch.) Babc. For *Nevskia* and *Herbaspirillum*, in addition to influencing plant nutritional metabolism. *Nevskia* typically plays a distinct role in the rhizosphere metabolic process, thereby affecting the accumulation of protostane triterpenes. This was also observed in *C. napifera* (Franch.) Babc. as the altitude gradually increased [38,39,40]. However, numerous reports have indicated that *Nevskia* primarily functions in the degradation of organic pollutants, particularly aromatic compounds [41,42]. Consequently, an intriguing hypothesis could be proposed: If it is confirmed that *C. napifera* (Franch.) Babc. exerts a recruitment effect on *Nevskia*, then, in addition to its inherent medicinal value, *C. napifera* (Franch.) Babc. could also serve as a phytoremediation agent for environmental pollution during the ecological restoration of contaminated areas. *Herbaspirillum*, an important endophytic bacterium that played a crucial role in plant nitrogen fixation, as well as in respiration, carbon metabolism, and cell wall metabolism, has also been reported in the literature [43,44]. Similarly, under varying altitudinal conditions, *C. napifera* (Franch.) Babc. exhibited distinct patterns in the recruitment of *Herbaspirillum*, which reflected the differential transcriptional expression and metabolite accumulation of *C. napifera* (Franch.) Babc. across different regions.

In the realm of drug development, natural products derived from plants, animals, and microorganisms are pivotal, as they represent key sources of lead compounds for therapeutic drug formulations [45,46]. Terpenes have emerged as one of the most promising categories of natural products, attributed to their diverse structural configurations and extensive range of types. These activities encompass anticancer effects, the ability to reverse multidrug resistance, and antiviral properties [44]. Furthermore, secondary metabolites hold a crucial position in determining the composition and functionality of the rhizosphere microbiome. They can act as signaling molecules, attract or repel specific microbes, and even modulate the metabolic activities of the rhizosphere microbiome [45,46,47,48,49]. The triterpenes present in the genus *Crepis*, as key bioactive compounds, play a crucial role in the efficacy evaluation [50]. For *C. napifera* (Franch.) Babc. populations distributed across various regions, we not only observed differences in the transcriptional expression of terpenoids and their metabolite accumulation processes but also noted with interest that the rhizosphere microbial communities exhibited variations correlated with differences in terpenoid accumulation. These specific microbial communities may function as natural defense mechanisms for plants, aiding in the prevention of pathogenic microbe invasion and maintaining a healthy rhizosphere microbial ecosystem. Furthermore, they can also serve as communication intermediaries between plants and beneficial microbes, fostering symbiotic relationships and thereby enhancing plant growth and health [51,52].

In summary, the accumulation of terpenoids in *C. napifera* (Franch.) Babc. across different regions is influenced by transcriptional level changes. Throughout the process of metabolite accumulation, rhizosphere microbial communities also undergo alterations, demonstrating a recruitment effect. The differential enrichment of specific microbial communities can further enhance plant growth and offer potential utilization value, thereby providing a scientific foundation for the exploration of this significant regional medicinal plant resource. Although we drew on such a multi-omics analysis on different altitudes of *C. napifera* (Franch.) Babc. to understand the survival strategy in alpine areas, there are still many issues of concern, such as the transcriptomic and accumulation mechanisms of terpenoids biosynthesis and the microbial community structure transform pattern during the vegetative period. More evidence is needed to explain how and why this plant is distributed in such a narrow area compared to the other genera in the Asteraceae family.

## 5. Conclusions

In this study, we conducted a comprehensive multi-omics analysis of the roots of *C. napifera* (Franch.) Babc. collected from three distinct alpine altitudes. Morphological assessments indicated significant alterations in the physical characteristics, including root length, fresh weight, pH value, carbon content, and density, as the altitude increased. The WGCNA revealed that the gene expression levels in the turquoise and yellow modules were positively correlated with elevation. Additionally, transcriptomic and metabolomic analyses demonstrated an increase in the accumulation of terpenoid metabolites in the roots of *C. napifera* (Franch.) Babc. with the rising altitude. The analysis of the microbial community further revealed that changes in the environmental conditions significantly influenced the composition of the microorganisms. Alterations in the transcriptional levels and variations in metabolite accumulation resulted in a pronounced recruitment effect within the rhizosphere microbial community. This recruited microbial community subsequently facilitated the accumulation of plant metabolites, particularly terpenoids, thereby enhancing plant growth. Evaluating *C. napifera* (Franch.) Babc. from diverse regions using multi-omics approaches has deepened our understanding of this medicinal plant and expanded its potential applications.

## Figures and Tables

**Table 1:** The environmental information of the sampling sites.

Sample Name	Altitude (m)	Location	Annual Average Air Temperature (°C)	Annual Precipitation (mm)	Average Temperatures of the Warmest Months (°C)	Average Temperatures of the Coldest Months (°C)
H1	1858	Jin Kedu, Xingming Xingyuan Village, Longpan Township	15.7	790.5	21.1	8.2
H2	2417	Le Shibanman, Xingwen Qiping Village, Longpan Township	12.2	839.1	18.2	6.4
H3	2893	Jizi Village, Tai’an Township	8.7	643.3	13.5	3.6

**Table 2:** Main characteristics of the studied soils from the rhizosphere of *C. napifera* (Franch.) Babc.

Sample Name	Soil Type	Available P (μg/g)	Available K (mg/kg)	pH Value	Total C (g/kg)	Total N (g/kg)	C:N
H1	red clay	6.04	512.91	6.52	13.63	1.62	8.46
H2	red clay	5.80	341.43	6.01	13.81	1.26	16.45
H3	Yellow sandy soil	5.93	250.46	6.35	15.22	1.40	11.73

## Data Availability

The data presented in this study are available on request from the corresponding author. The data are not publicly available due to privacy.

## Supplementary Materials

The following supporting information can be downloaded at https://www.mdpi.com/article/10.3390/biology14040345/s1: Figure S1: WGCNA and expression trend analysis; Figure S2: Differentially expressed metabolites classification and cluster analysis; Figure S3: Heatmap of the abundance of microbial communities at the genus level; Figure S4: Schematic of the metabolism and genes involved in the terpenoid biosynthesis at different altitudes; Table S1: Primers used for qRT-PCR in this study; Table S2: Phenotype statistics under different altitude conditions; Table S3: Quality control of the sequencing results; Table S4: Statistics of differentially expressed genes; Table S5: GO analysis of the genes obtained through WGCNA; Table S6: KEGG analysis of the enriched metabolism.

## Abbreviations

The following abbreviations are used in this manuscript:

16S rRNA	16S ribosomal RNA
DEGs	Differentially expressed genes
DEMs	Differentially expressed metabolites
UV	Ultraviolet rays
UPLC-MS/MS	Ultra performance liquid chromatography–tandem mass spectrometry
MRM	Multiple reaction monitoring
OPLS-DA	Orthogonal partial least squares discriminant analysis
RS	Rhizosphere soil
PCA	Principal component analysis
MSEA	Metabolite sets enrichment analysis
qRT-PCR	Quantitative real-time polymerase chain reaction
WGCNA	Weighted gene co-expression network analysis
KEGG	Kyoto encyclopedia of genes and genomes
