# Supplementary figures and images for: Alpine Adaptive Mechanism on Rhizosphere Microbes Recruitment of Crepis napifera (Franch.) Babc. by Multi-Omics Analysis
Source: Biology (Basel). 2025 Mar 27;14(4):345. doi: 10.3390/biology14040345 (PMC12025305; doi:10.3390/biology14040345)

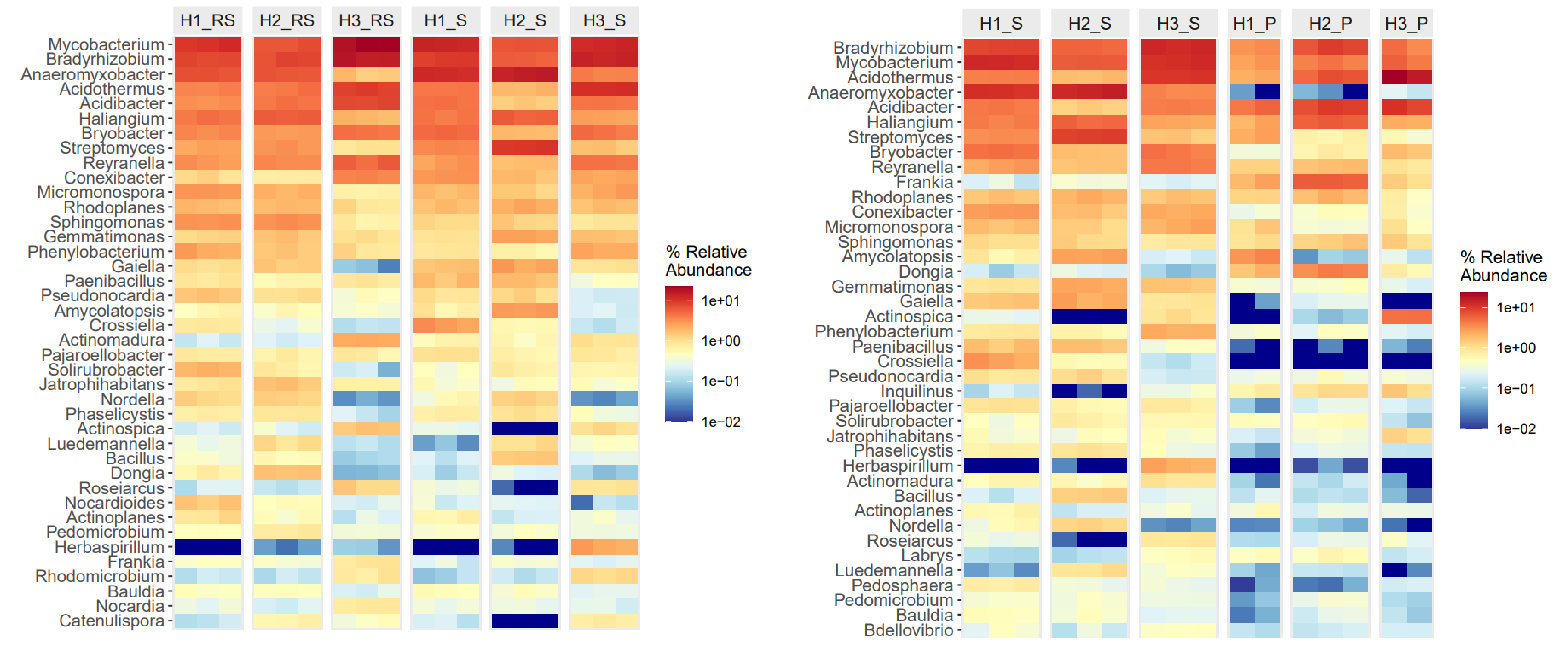

Supplement: Supplementary file 1 [file biology-14-00345-s001.zip › Figure S3 Heatmap of abundance of microbial communities at genus level.tif]

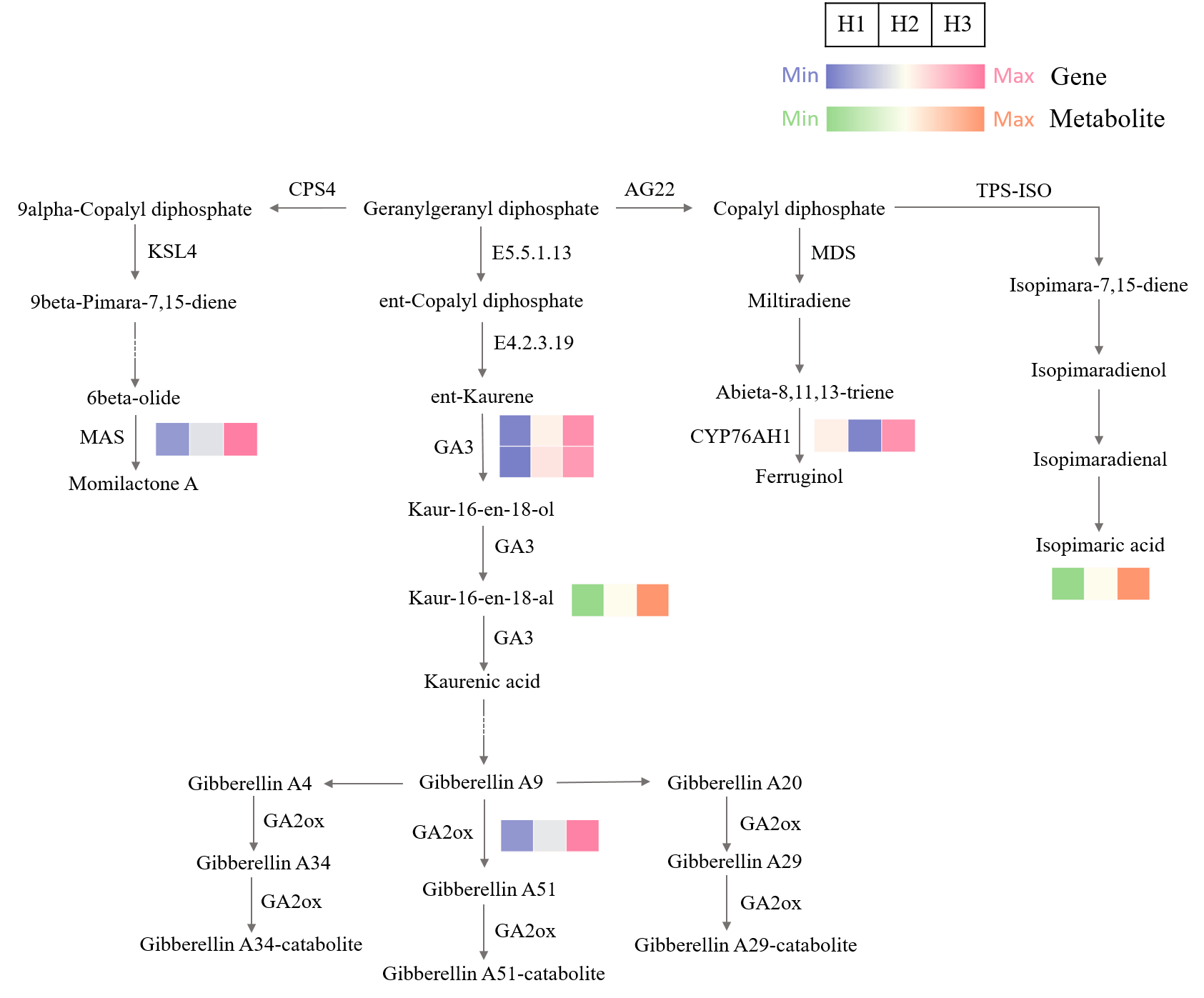

Supplement: Supplementary file 1 [file biology-14-00345-s001.zip › Figure S4 Schematic of metabolism and genes involved in the terpenoid biosynthesis at different altitude.tif]

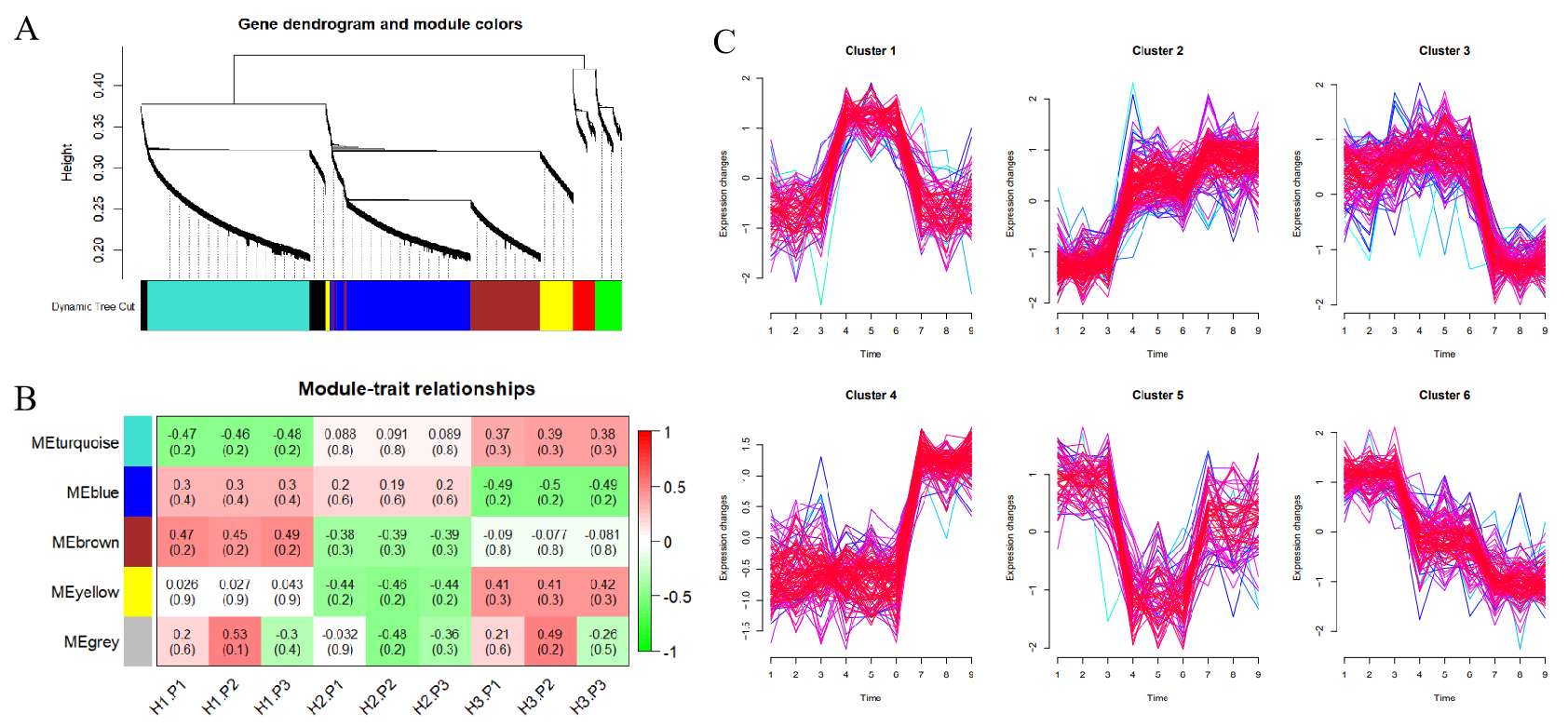

Supplement: Supplementary file 1 [file biology-14-00345-s001.zip › Figure S1 WGCNA analysis and expression trend analysis.tif]

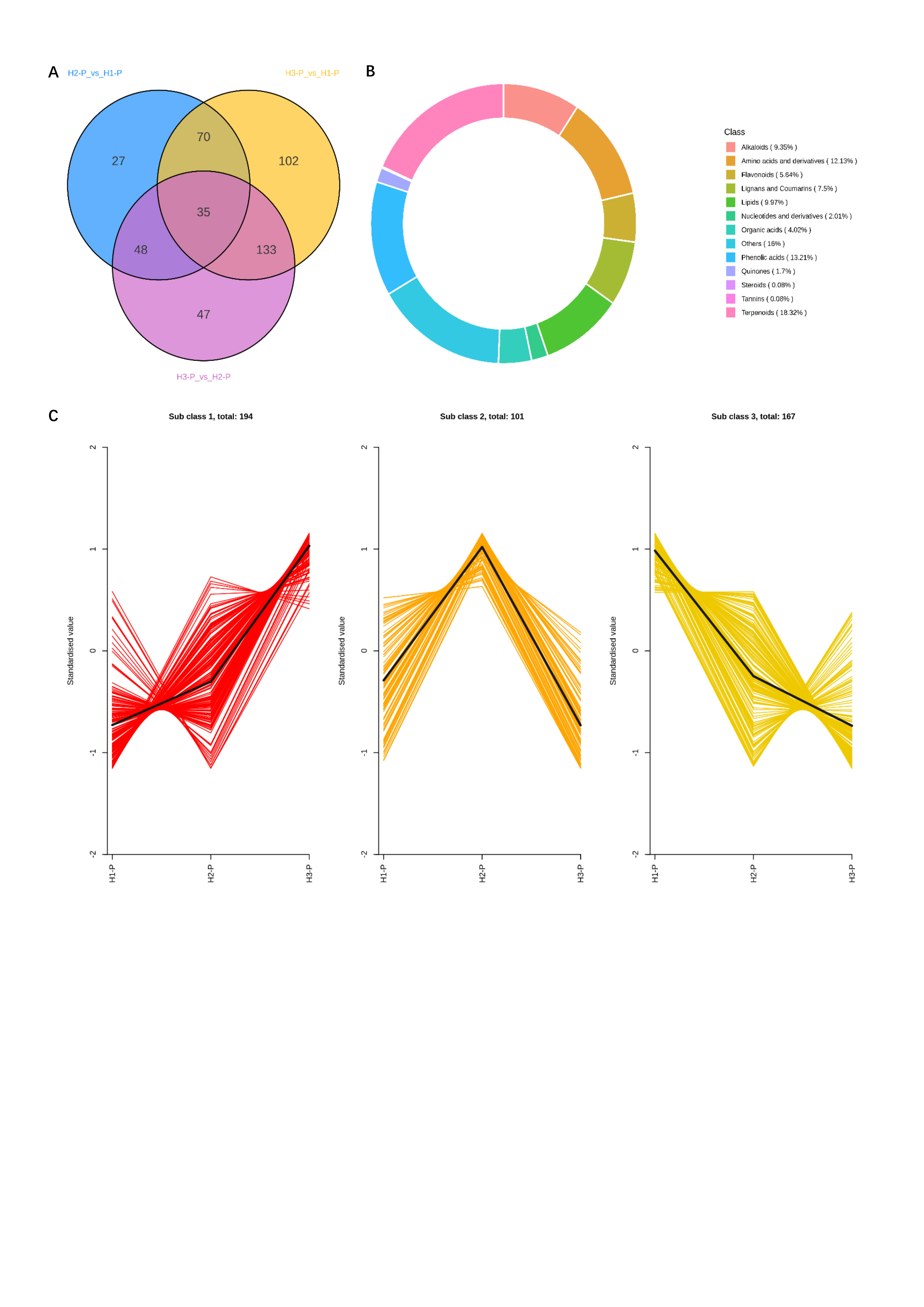

Supplement: Supplementary file 1 [file biology-14-00345-s001.zip › Figure S2 Differentially expressed metabolites classification and cluster analysis.jpg]
